# Effect of Fabric Topology and Axial Yarn Condition on the Compressive Properties of 3D Stepwise Rotary Braided Composites

**DOI:** 10.3390/ma18245561

**Published:** 2025-12-11

**Authors:** Haiyang Mei, Long Sun, Ran Yang, Qian Zhang, Yankuo Guo, Zhenyu Han

**Affiliations:** 1 School of Mechanical and Automotive Engineering, Qingdao University of Technology, Qingdao 266520, China; 2 School of Intelligent Manufacturing, Qingdao Huanghai University, Qingdao 266427, China; 3 School of Mechatronics Engineering, Harbin Institute of Technology, Harbin 150001, China

**Keywords:** three-dimensional braided composites, compressive properties, stepwise rotary braiding, fabric topology, axial yarn condition

## Abstract

Three-dimensional braided composites have become one kind of critical engineering material for applications in extreme environments. The 3D stepwise rotary braiding process is one vital technique for manufacturing preforms with high efficiency and flexibility. However, the fabric topology is decided by the combination of switch rotation directions, which affects the mechanical properties, and the full carrier configuration results in a loose four-directional structure which is supposed to be improved by adding axial yarns. Therefore, experiments are carried out to illustrate the effect of fabric topology and axial yarn condition on the compressive properties of 3D stepwise rotary braided composites. Samples with three types of fabric topologies named Type A, B, and C are prepared under four axial yarn conditions including no axial yarn addition, 12K axial yarn addition, 24K axial yarn addition, and 36K axial yarn addition, which are fabricated with braiding angles of 20°, 30° and 40°. Longitudinal and transverse compression tests are conducted, and the morphology is observed. It shows that the braiding angle has more influence on the longitudinal compressive properties than transverse compressive properties, and the effect of fabric topology and axial yarn condition depends on the braiding angle. The fabric topology affects a lot on the longitudinal compressive properties when the braiding angle is small, resulting in a gap of up to 40%. The longitudinal compressive properties are improved significantly by adding axial yarns especially for the composites with large braiding angles, making the strength more than double. With the increase in axial yarn size, the strength increment gradually decreases while the modulus declines after a certain size for smaller braiding angles.

## 1. Introduction

Three-dimensional braided carbon fiber reinforced composites have received great attention in aerospace, aeronautic and automotive industries due to their excellent mechanical properties such as impact tolerance and energy absorption [1,2]. The compressive property is a key factor for applications in specific circumstances for load-bearing components. In order to make better use of the designability of braided structures, many studies have been focused on the structure–property relationship under compression.

Regarding 3D four-directional braided composites, braiding angle is the decisive parameter for longitudinal compressive properties but affects the transverse compressive properties less [3,4]. There is supposed to be a critical braiding angle for the longitudinal compression failure mechanism, below which the material exhibits more brittleness, and above which it exhibits more plasticity. It is observed that the fiber bundle fracture is the main failure mode for samples with small braiding angle, while the delamination at the fiber bundle/matrix interface is the main failure mode for samples with large braiding angle [5]. Additionally, the longitudinal compressive strength and modulus become higher when the braiding angle becomes smaller. Fang et al. [6] found that the compressive behavior of the composites with lower braiding angle is sensitive to the fiber’s initial imperfection. Zhu et al. [7] presented the effects of braiding angle, fiber volume fraction and loading direction on the uniaxial compressive progressive damage and predicted the effective compressive modulus and strength. Yan et al. [8,9] studied the effect of braiding angle on the hygroscopic behavior and the effect of hygrothermal aging on the mechanical properties in the compression after impact tests. Wang et al. [10] illustrated the difference in mechanical responses and porous damage tolerance resulting from different braiding angles and different types of prefabricated holes. Gu et al. [11,12,13,14,15] systematically investigated the dynamic compressive behaviors with different braided structures, loads and environments.

With stationary yarn addition, the 3D multi-directional braided composites exhibit various performances [16]. Liang et al. [17] found that the introduction of axial yarns has limited effect on improving compressive strength for composites with small braiding angles. Cao et al. [18] showed that the compressive performance of 3D full five-directional composites is better than that of 3D five-directional composites when the braiding angle and fiber volume fraction are similar. Zuo et al. [19] illustrated that the 3D five-directional braided composites with smaller braiding angles always have longer fatigue lives, although their failure modes under longitudinal and transverse compression loads are different. Chen et al. [20] indicated that the 3D five-directional and six-directional braided composites show the brittle failure mode in the longitudinal compression test, while the six-directional structure has lower compressive strength, compressive modulus, and Poisson’s ratio. In addition, reducing the fineness of braiding yarns can increase the volume ratio of in-plane yarns in the five-directional and six-directional composites, thereby effectively improving the in-plane performance. Zhu et al. [21,22] illustrated the influence of temperature and loading direction on the failure modes of 3D six-directional braided composites under in-plane and out-of-plane compressions. Wang et al. [23,24] investigated the effect of D/t ratio on 3D six-directional braided composite tubes under lateral compression and revealed the role of the sixth-directional yarn.

The 3D stepwise rotary braiding process is one promising technique to fabricate preforms for 3D braided composites with high efficiency and flexibility [25,26], accompanied by two issues with fabric structures. First, the combination of switch rotation direction decides the fabric topology, which affects the straightness of yarns in the composites and is supposed to affect the mechanical properties [27]. Second, the full carrier configuration leads to a loose four-directional braided structure which decreases the fiber volume fraction and makes yarns easy to deform, resulting in poor mechanical properties [28]. However, there are no references addressing these two issues [29,30,31,32,33].

Therefore, this paper aims to reveal the effect of fabric topology and axial yarn condition on the compressive properties of 3D braided composites made from the stepwise rotary braiding process through experiments. The rest of this paper is organized as follows. Section 2 introduces the design and fabrication of samples. Section 3 depicts the test results. Section 4 illustrates the effect of fabric structures. Conclusions are summarized in Section 5.

## 2. Materials and Methods

### 2.1. Fabric Design

Regarding four-directional preforms manufactured from the 3D stepwise rotary braiding process, the combination of switch rotation direction and carrier configuration jointly determine the fabric topology. In this paper, the full carrier configuration is utilized; i.e., all possible carrier positions are occupied. In this case, the difference in the combinations of switch rotation direction decides the fabric topology. Only under certain fabric topologies can the braiding yarns inside the fabric remain straight [27].

**Figure 1:**
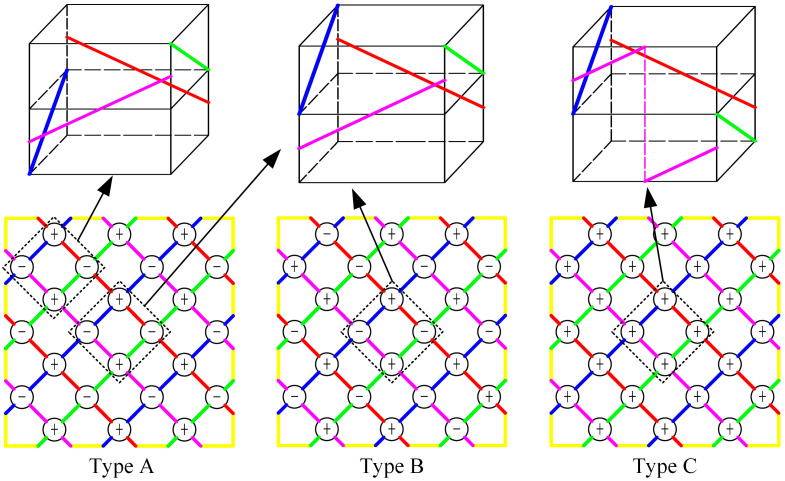
Fabric topologies described with the combination of switch rotation direction (“+” represents the clockwise switch rotation, and “−” represents the counterclockwise switch rotation).

According to the relationship between yarn topology and the combination of switch rotation direction, three types of fabrics are designed, which are marked as Type A, Type B and Type C. Fabrics of Type A and B have a topology with the potential to make braiding yarns inside the fabric remain straight, while fabrics of Type C have a topology which cannot keep yarns straight. The switch rotation direction combinations are shown in Figure 1, in which “+” represents clockwise switch rotation and “−” represents counterclockwise switch rotation. In the fabrics of Type A, red and purple yarns in the upper left corner and the lower right corner interlace with other yarns at a higher position, and at a lower position when interlacing at other locations. In the fabrics of Type B, red and purple yarns are always at a lower position during each interlacement. Regarding the fabrics of Type C, all switches rotate clockwise, meaning yarns cannot keep straight. As to the manufacturing of fabrics of these three topologies, the same horn-gear rotation movement is used and the difference lies in the switch rotation movement, which is kept consistent in the braiding process.

Since the four-directional fabrics with full carrier configuration have a loose structure, in which the braiding yarns tend to bend due to the inherent axial gap providing deformation space [28], axial yarn addition is considered to be an effective means of filling the gap, thus helping braiding yarns keep straight. In this paper, axial yarns of 12K, 24K and 36K are adopted to form 3D full five-directional braided fabrics.

### 2.2. Sample Fabrication

The carbon yarns of different sizes belong to the TZ700S series of Weihai Guangwei Co., Ltd. of China (Weihai, China), and the main parameters are shown in Table 1.

**Figure 2:**
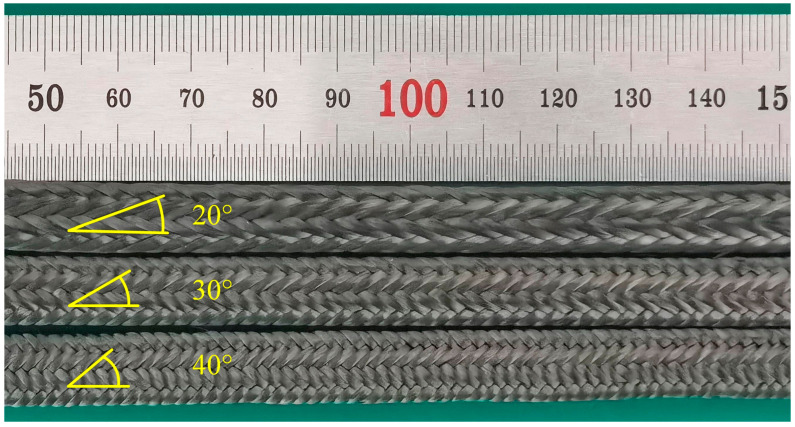
Preforms with different braiding angles.

The braided preforms are fabricated on a stepwise 3D rotary braiding machine with braiding angles of 20°, 30° and 40°, which are shown in Figure 2.

**Figure 3:**
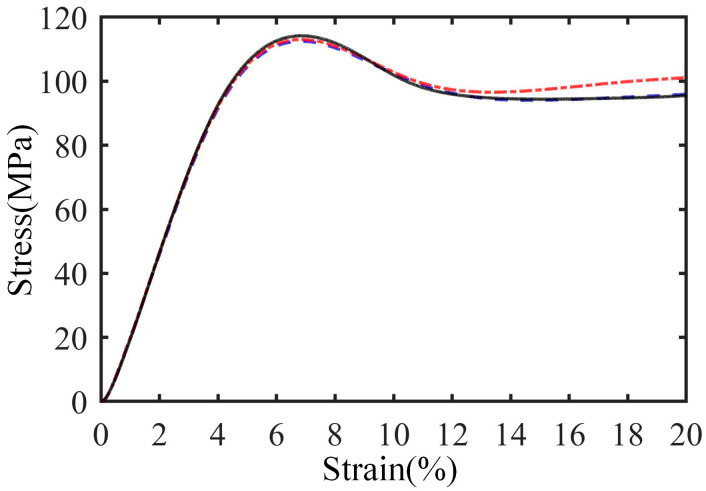
Stress–strain curves of matrix samples.

The matrix is a two-part casting epoxy (from Huzhou Hangmo Co., Ltd. of China, Huzhou, China) comprising JC-02A and JC-02B, mixed 100 parts to 85 parts by weight. The properties of the matrix are shown in Table 2, in which the values are calculated from the average of three tests. The stress–strain curves of these three matrix samples are shown in Figure 3.

The preforms are cut and put into a die which connects with the resin and vacuum device by piping. Then, the matrix is injected into preforms with a vacuum-assisted resin transfer molding instrument at −0.1 MPa to prepare the braided composite. The curing process is 2 h at 90 °C, 1 h at 110 °C and 4 h at 130 °C. After consolidation, the composites are cut to be of specific lengths.

The specification of each kind of composite is shown in Table 3. The naming method of the composites is “T-AY-BA”, where “T” represents the fabric topology, “AY” is the axial yarn size, in which 0 represents the condition without axial yarn, and “BA” represents the braiding angle. All kinds of composites are subject to longitudinal compression tests while eight kinds of samples are selected for transverse compression tests, as marked in Table 3. The fiber volume fraction is measured by the weighing method. It can be seen that the fiber volume fraction of these three types of composites with different fabric topologies has little difference when the braiding angle is the same. However, the fiber volume fraction is closely related to the axial yarn condition, which varies across a large range from approximately 40% to 60%.

### 2.3. Experimental Procedure

**Figure 4:**
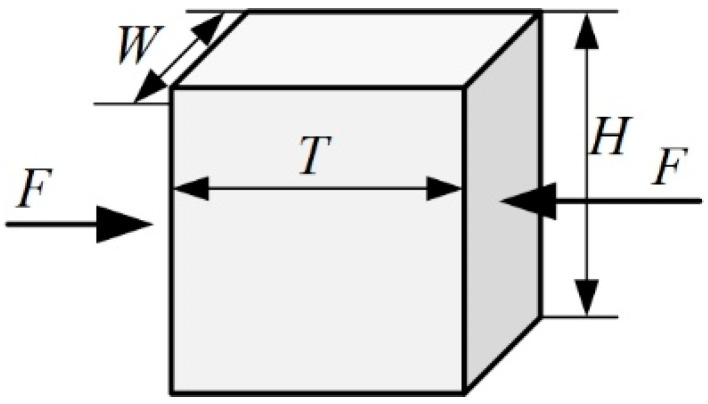
Shape, size and loading direction of transverse compressive samples.

According to the GB/T 1448-2005 standard [34], the samples used in the longitudinal compressive tests are machined to 30 mm high, while the height of samples used in the transverse compressive tests is 10 mm, which can guarantee that the failure is governed by material damage other than Euler buckling. In order to ensure the consistency of results, the transverse compression load is applied in the thickness direction, as shown in Figure 4.

All samples are tested on a WDW-100 computer-controlled electronic universal testing machine at a tension rate of 1 mm/min at room temperature, and three samples are utilized in each test. Before the test, the ends of each sample are milled to ensure that the end faces are perpendicular to the length direction. In the tests, samples are placed in the medium between two platens, and the corresponding load and displacement of the platens are recorded by the instrument.

**Figure 5:**
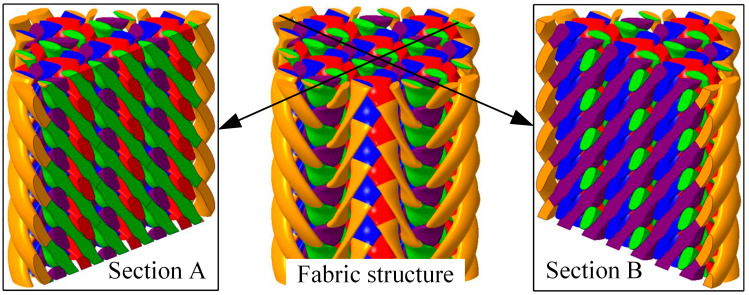
Sections to show the yarn morphology.

In order to depict the effect of fabric topology and axial yarn condition on the fabric structure, the yarn morphology in composites of 30° braiding angle is observed along the direction of 45° with the surface of the samples. Due to the differences in the morphology between interlaced yarns, the samples are cut along two vertical planes, which is shown in Figure 5. Then, the section is polished and photographed using a high-resolution camera.

## 3. Results

### 3.1. Yarn Morphology in Composites

**Figure 6:**
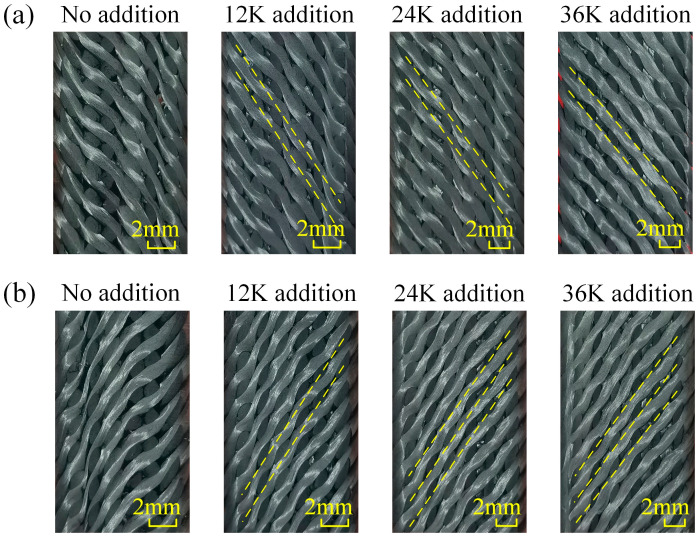
Yarn morphologies in the samples of Type A: (**a**) morphology of Section A, (**b**) morphology of Section B.

The yarn morphology in samples of Type A is shown in Figure 6. It can be seen that when no axial yarn is added, yarns present a parabola shape on the whole, while bending in local areas, and there exist obvious width changes along yarns caused by the twist deformation. The cross-sectional shapes of interlaced yarns are quite different and the position distribution is uneven. After adding axial yarns, braiding yarns present regular wave shapes due to the extrusion from interlaced yarns, the consistency and uniformity of which are improved. A straight line can be obtained by connecting the geometric center of the yarn sections on both sides of the section, as shown by the dotted line. Upon comprehensive comparison, it is seen that the straightness of braiding yarns increases with the increase in the axial yarn size, and the local bending is reduced simultaneously. When the axial yarn size is 36K, the braiding yarns tend to be straight lines, except for the inevitable local bending caused by the mutual extrusion.

**Figure 7:**
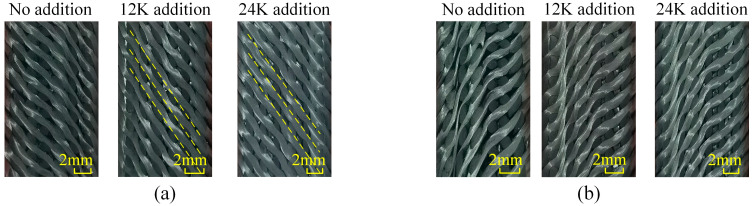
Yarn morphologies in the samples of Type B: (**a**) morphology of Section A, (**b**) morphology of Section B.

Similar to the samples of Type A, the consistency and uniformity of yarns in samples of Type B are poor when there is no axial yarn addition, which is shown in Figure 7, and yarns in both sections are in the shape of parabolas. With the addition of axial yarns, the consistency and uniformity of the morphology are improved, and the straightness of yarns on Section A is increased. When the axial yarn is 24K, the braiding yarns are approximately straight, the change in cross section is small, and the bending fluctuation is reduced. It is worth noting that the braiding yarns on Section B do not straighten with the addition of axial yarn, but continue to maintain their parabolic shape. Comparing the yarn morphology in samples of Type A and B, it can be seen that although they have the same internal interlacing structure, there are great differences in the yarn morphology due to the difference in topology, which further shows that they are two completely different types of fabrics.

**Figure 8:**
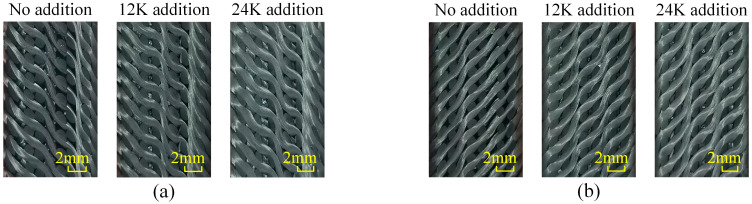
Yarn morphologies in the samples of Type C: (**a**) morphology of Section A, (**b**) morphology of Section B.

As shown in Figure 8, the change in yarn morphology in samples of Type C shows a different rule from the above two types. When there is no axial yarn added, the structure also has good consistency. Yarns in Section A are stepped on the whole and the morphology hardly changes with the axial yarn condition. The grid formed by the steps divides the cross-sections of the six yarns passing through the section into a “3 + 2 + 1” distribution. Regarding Section B, yarns also present an obvious ladder shape after adding axial yarn, which divides the cross sections of yarns passing through the section into a “2 + 2 + 2” distribution; however, this characteristic is not obvious when no axial yarn is added.

The above observation results show that the axial yarn can improve the consistency and uniformity of the reinforcement in composites of Type A and B, which may be due to the fact that the axial yarn reduces the deformation space of braiding yarns and provides support as well. In these two types of composites, the yarn morphology gradually tends to be stable with the increase in axial yarn size. As for the composites of Type C, the addition of axial yarn does not have an obvious effect, which may be due to the fact that braiding yarns are already twisted due to the unitary switch rotation directions during the manufacturing process, thus reducing the deformation ability. Also, the results prove the deduction in reference [27] that the topology is just a necessary condition to keep the straightness of yarns and there are unknown laws governing it. The ranking of yarn straightness is qualitative, which cannot provide data support. Quantitative morphology analysis is warranted for discovering the statistical relations in the future.

### 3.2. Longitudinal Compressive Properties of 3D Braided Composites

The longitudinal compressive properties are shown in Table 4.

**Figure 9:**
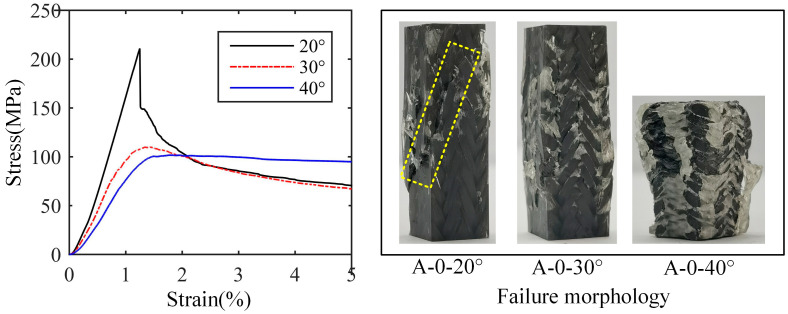
Stress–strain curve and failure morphology of longitudinal compression samples without axial yarn of Type A.

The stress–strain curve and failure morphology of longitudinal compression samples without axial yarn of Type A are shown in Figure 9. It can be seen that the compression load of the samples of 20° braiding angle drops sharply after reaching the maximum value, which indicates brittle failure. There is an obvious macro shear failure on the sample, and the shear failure crack is along the direction of the smaller surface braiding angle. Regarding the samples of 30° or 40° braiding angle, there are obvious yield points and nonlinear stages in the stress–strain curves, which suggests plastic failure. In the samples of 30° braiding angle, the fiber bundle bends and the matrix cracks. There are three cracks along the height direction of the sample, passing through the interlacing point between surface yarns; this may be caused by the yarns of different groups bending towards different directions under the compression load. Then, the fiber bundle gradually breaks, resulting in the slow decline of the bearing capacity. For the samples of 40° braiding angle, the bearing capacity decreased slightly after the yield stage, and then increased slowly due to the compaction of the material. Compared with the samples of 20° and 30° braiding angles, the deformation up to the fiber bundle fracture of 40° braiding angle increases significantly. An interesting phenomenon is noted that the samples of 40° braiding angle have a fairly long plastic stage with considerable bearing capacity.

**Figure 10:**
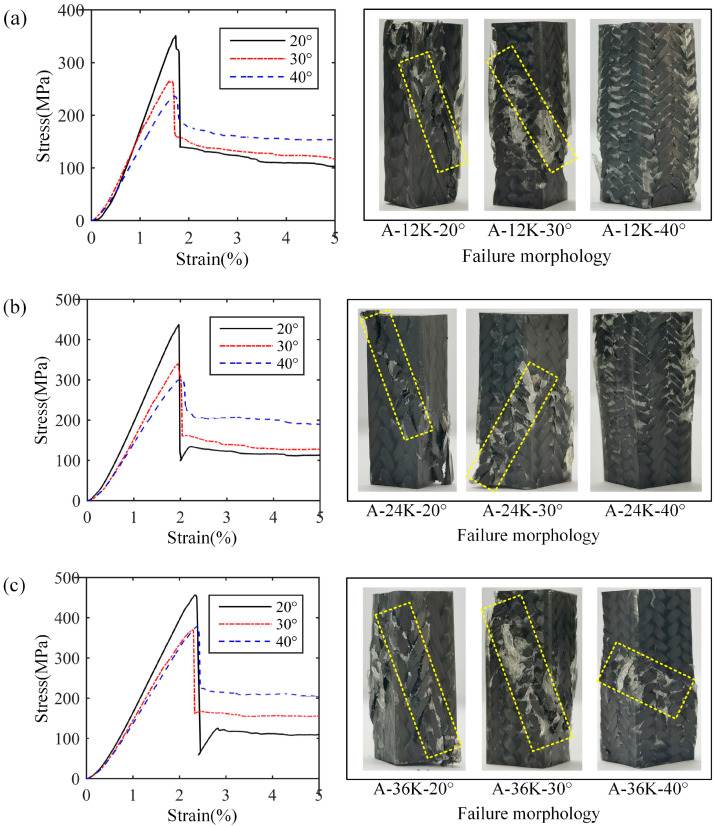
Stress–strain curves and failure morphologies of longitudinal compression samples with axial yarns of Type A: (**a**) results with 12K axial yarn, (**b**) results with 24K axial yarn, (**c**) results with 36K axial yarn.

The stress–strain curves and failure morphologies of longitudinal compression samples with axial yarns of Type A are shown in Figure 8, in which Figure 10 represent the results with axial yarns of 12K, 24K and 36K, respectively. It is noted that when the axial yarn is added, the samples show brittle failure no matter the braiding angle. Compared with the samples without axial yarn, the failure morphology of samples of 30° braiding angle changes significantly, with obvious cracks appearing on the surface of the sample, which suggests macro shear failure. When the axial yarn size is 12K and 24K, the samples of 40° braiding angle expand into a drum shape, and the surface fiber bundle does not appear obvious fracture when it fails. However, when the axial yarn size is 36K, an obvious shear resin band occurs, which the surface yarns break and protrude through, indicating that although the samples of 40° braiding angle have strong shear resistance, the internal crack can also extend to the surface when the failure load is large enough, resulting in the surface yarns being cut to form a through crack.

**Figure 11:**
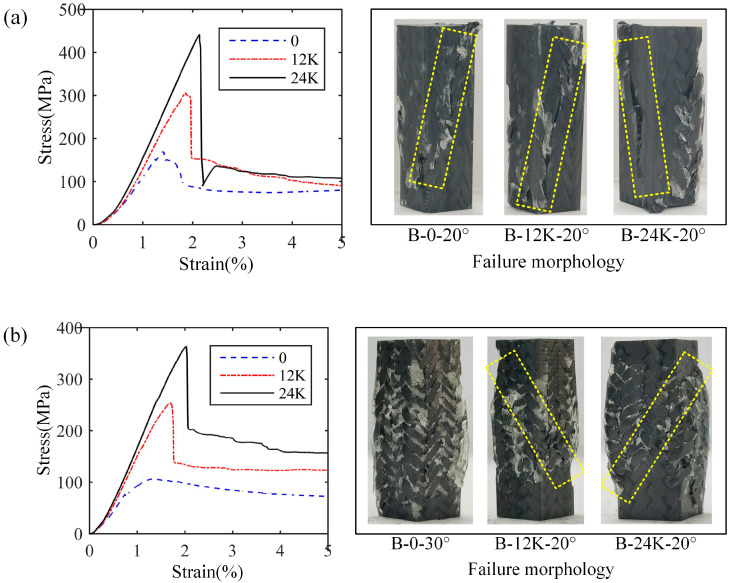
Stress–strain curves and failure morphologies of longitudinal compression samples of Type B: (**a**) results with 20° braiding angle, (**b**) results with 30° braiding angle.

Figure 11 shows the test results of samples of Type B, in which Figure 11a,b represent the results with braiding angles of 20° and 30°, respectively. It can be seen that the addition of axial yarn significantly improves the bearing capacity of the composites, which is similar to the samples of Type A. However, the samples of 30° braiding degree without axial yarn of Type B indicates a ductile failure mode, which is different from that of Type A. All samples containing axial yarns suggest obvious shear failure, no matter the braiding angle, which illustrates the importance of the axial yarn addition.

**Figure 12:**
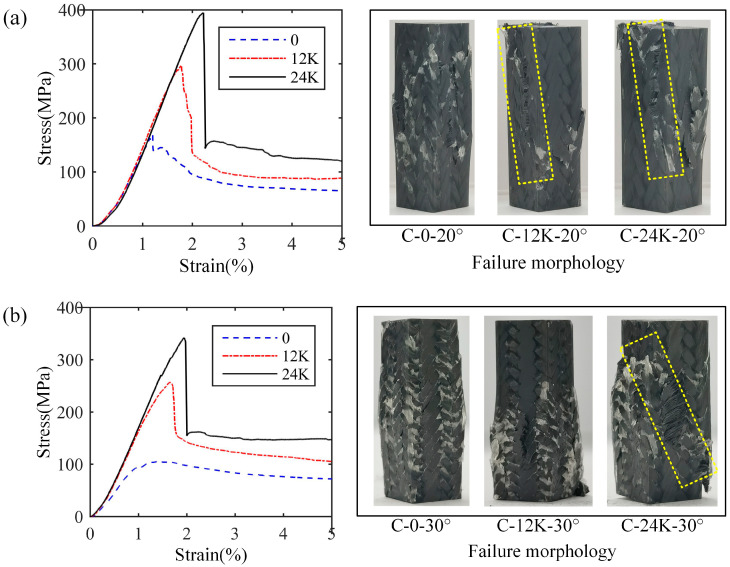
Stress–strain curves and failure morphologies of longitudinal compression samples of Type C: (**a**) results with 20° braiding angle, (**b**) results with 30° braiding angle.

The situation of the samples of Type C is a little different from that of Type B, which is shown in Figure 12. For the samples of 30° braiding angle without axial yarn, irregular cracks appear on the surface instead of the shear band similar to that of Type A and Type B. Meanwhile, regarding the samples of 30° braiding angle without axial yarn and with 12K axial yarns, the expansion of the sample leads to the bending and breaking of the surface yarns without local shear characteristics. These may be attributed to the fact that the serious step-like local bends of the yarns in the composites would prevent crack propagation. At the same time, the stress of the samples of 30° braiding angle without axial yarn decreases briefly before reaching the maximum value, which is the same as that of the samples of another two types, and there is a certain non-linear segment. This may be due to the fact that the crack is difficult to expand along the yarns with complex deformation; the bearing capacity thus recovers based on a new load transfer route in the composites with compaction of the sample.

### 3.3. Transverse Compressive Properties of 3D Braided Composites

**Figure 13:**
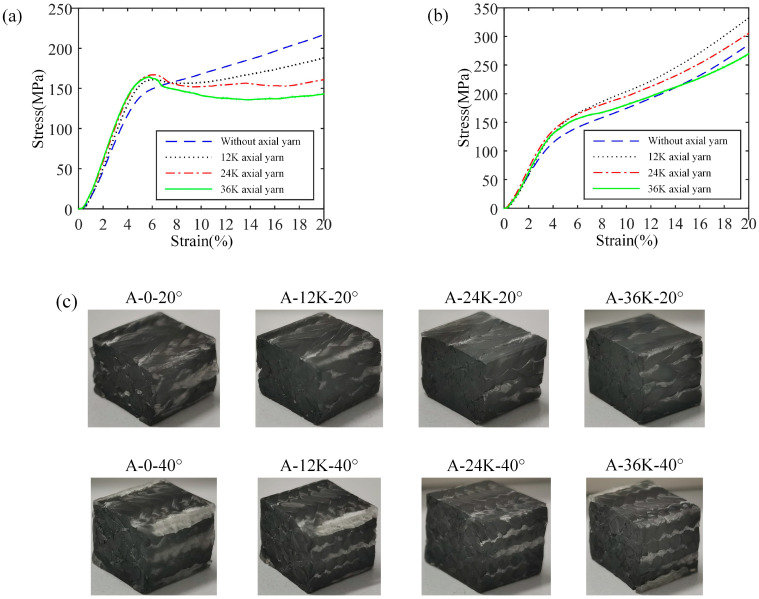
Stress–strain curves and failure modes of transverse compression samples: (**a**) stress–strain curves of samples with 20° braiding angle, (**b**) stress–strain curves of samples with 40° braiding angle, (**c**) failure morphologies.

The stress–strain curves and failure morphologies of the transverse compression samples are shown in Figure 13. It can be seen that the samples are flattened and the surface resin is broken due to the extrusion under transverse compression load. This is attributed to the complete integrated surface and corner structure, and there is no shear band or yarn dispersion. All samples show an obvious elastic stage and plastic stage, and the stress and strain change linearly in the elastic stage. The addition of axial yarn increases the fiber volume fraction of the composite, thus improving the modulus. Therefore, the modulus is not sensitive to the change in axial yarn size. At the same time, after adding axial yarn, the stress–strain curve of the samples with 20° braiding angle has an obvious descent stage generally seen in the brittle failure mode, which may be caused by two reasons. First, the braided composites with 20° braiding angle have a large pitch length; as such, yarns in the samples have less interlacement, resulting in fewer local bends. Second, the degree of local bending of yarns is reduced after adding axial yarns, which makes the crack generated by the debonding between the yarn and the matrix easy to expand. On the contrary, the pitch length of the composites of 40° braiding angle is small; as such, most yarns are located between different surfaces, which provides more support, and the samples have more interlacement, resulting in serious local bends making the crack difficult to expand. From the failure morphology of the samples, it can be seen that the cracks of samples of 20° braiding angle are concentrated and expand into multiple lines on the cross-section, while the cracks of samples of 20° braiding angle are scattered. It is worth noting that for the samples of 20° braiding angle without axial yarn, there occurs a near-linearity stage after the first elastic stage in the stress–strain curve, which is similar to linear strengthening material.

The transverse compressive properties are shown in Table 5, in which the stress with 0.2% plastic strain in the curves of samples of 20° braiding angle without axial yarn and 40° braiding angle are taken as the strength. It can be seen that the modulus of samples of the two braiding angles are relatively close, indicating that the braiding angle has little effect on the transverse compression modulus. When 12K axial yarn is added, the compression modulus is significantly improved, while the change in axial yarn size has little effect on it, which indicates that the fiber volume fraction has a greater impact on the transverse compression modulus. Also, the increase in its size has little effect on the compressive strength, which may be attributed to the fact that the axial yarn is perpendicular to the load direction.

## 4. Discussion

The above results show that the longitudinal compressive properties are affected by the braiding angle, axial yarn condition and fabric topology. Properties in a wide range (for the strength from 100.17 MPa to 447.55 MPa, for the modulus from 9.78 GPa to 25.27 GPa) can be obtained by adjusting these factors.

### 4.1. Effect of the Braiding Angle on the Longitudinal Compressive Properties

**Figure 14:**
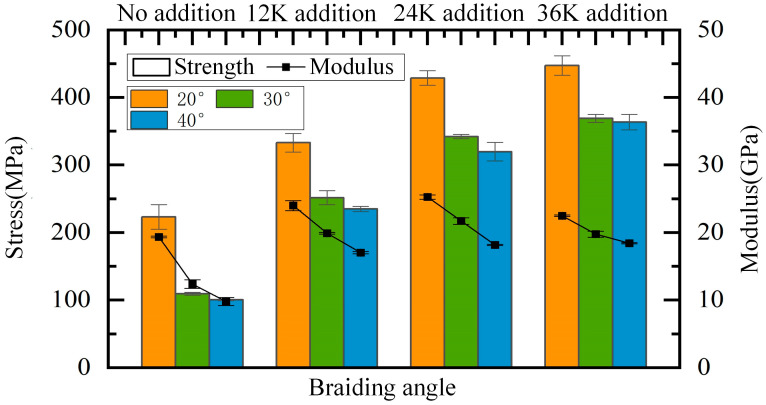
Effect of the braiding angle on longitudinal compressive properties.

As shown in Figure 14, the compressive strength and modulus decrease with the increase in the braiding angle when the axial yarn condition is the same, which is because the braiding angle represents the spatial direction of braiding yarns. When the braiding angle increases, the angle between braiding yarns and the loading direction increases, resulting in a decline in the resistance to deformation and damage. When there is no axial yarn added, the compressive strength of samples of 30° and 40° is 109.31 MPa and 100.17 MPa, respectively, which is slightly lower than that of pure matrix. Regarding strength performance, the fiber preform is more like an impurity than a reinforcement. This may be due to the large braiding angle which makes the impregnated fiber bundle easily overwhelmed under compression, and the load is mainly borne by the matrix. At the same time, the compressive strength of samples of 30° and 40° braiding angles is close to each other and far lower than that of 20° braiding angle under any condition of axial yarn addition, which indicates that the axial compressive strength does not change significantly with a large braiding angle. It can be concluded that the compressive strength of composites with a large braiding angle is mainly determined by the matrix when there is no axial yarn, and by the axial yarn when the axial yarn is added. After adding axial yarns, the difference between the compressive modulus of samples of 30° and 40° braiding angles and that of 20° braiding angle is significantly reduced, which also shows that the axial yarn bears most of the compression load in the samples of 30° and 40° braiding angles, and the failure is mainly caused by the fracture of axial yarns. With the addition of axial yarns and increase in the axial yarn size, the proportion of the load axial yarn bears increases; as such, the ratios of the compressive strength of samples of 20° braiding angle to that of 30° and 40° braiding angles decrease continuously.

### 4.2. Effect of the Axial Yarn Condition on the Longitudinal Compressive Properties

**Figure 15:**
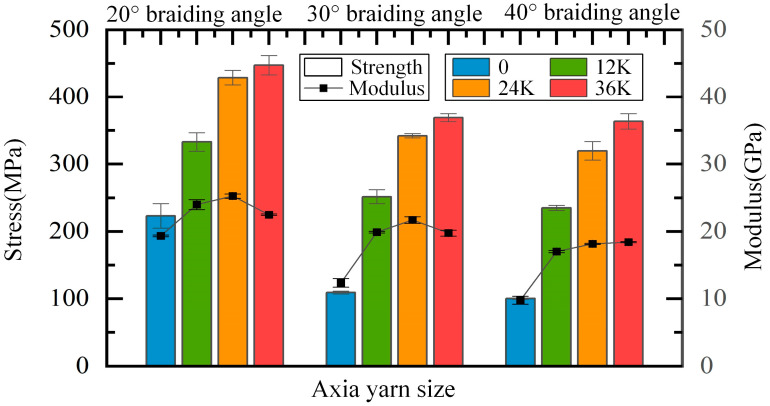
Effect of axial yarn condition on longitudinal compressive properties.

The effect of axial yarn condition on the longitudinal compressive properties of samples is shown in Figure 15. It can be seen that the addition of axial yarn significantly improves the compressive strength and modulus of composites, of which the values become more than doubled for composites of 30° and 40° braiding angles. When the axial yarn size increases, although the compressive strength gradually increases, the increment gradually decreases. Taking the samples of 30° braiding angle as an example, the strength increased by 142.38 MPa, 90.55 MPa and 26.79 MPa, respectively, from the addition of axial yarn to the increase in axial yarn size to 36K. This is mainly caused by the characteristics of the fabric’s structure. When the axial yarn size increases from 12K to 24K, the supporting effect of the axial yarn on braiding yarns is enhanced, the straightness of braiding yarns is improved, the axial gap through the four-directional fabrics is further filled, and the fiber volume fraction is improved, thus the performance is greatly improved. However, when the axial yarn size increases from 24K to 36K, and the axial yarn of large size stretches the braiding yarns and fills the gap, introducing new gaps and forming a new resin-rich area, and the fiber volume fraction decreases slightly. Therefore, the performance is less improved, although the portion of axial yarn volume is increased obviously. For composites of 30° and 40° braiding angles, the strength improvement obtained by adding 12K axial yarns is much greater than that obtained by increasing the axial yarn size by 12K. This is mainly because adding axial yarn to the four-directional fabric structure fills the inherent gap without introducing a new gap, which greatly improves the fiber volume fraction and reduces the pure resin area.

When the axial yarn size is increased from 12K to 24K, the modulus of the composite material is slightly improved. However, when the axial yarn size increases from 24K to 36K, the change in compressive modulus shows different rules with different braiding angles. The modulus of samples of 20° and 30° braiding angles slightly decreases, but that of 40° braiding angle increases slightly, which may be mainly due to the different size of the internal axial gap with different braiding angles. The smaller the braiding angle, the smaller the axial gap in the four-directional fabrics structure. Therefore, large axial yarns stretch the inherent fabric structure, resulting in a decrease in the fiber volume fraction, thus reducing the modulus. That is to say, a big axial yarn size does not mean a high performance in longitudinal compressive properties.

### 4.3. Effect of the Fabric Topology on the Longitudinal Compressive Properties

**Figure 16:**
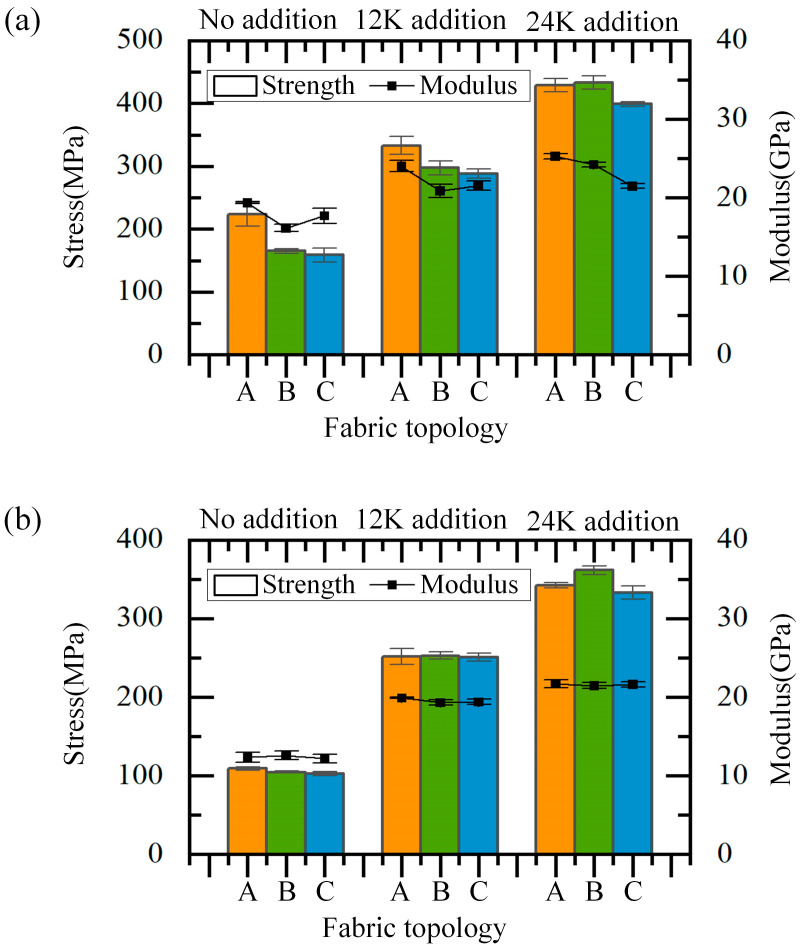
Effect of the fabric topology on longitudinal compressive properties: (**a**) the situation when the braiding angle is 20°, (**b**) the situation when the braiding angle is 30°.

The effect of fabric topology on the longitudinal compressive properties is shown in Figure 16. It can be seen that when the braiding angle is 20°, the strength of composites without axial yarn of Type A is significantly higher than that of Type B and C, which are 233.05 MPa, 164.96 MPa and 158.51 MPa, respectively. When the axial yarn is 12K and 24K, the strength difference between composites of Type A and B is 35.90 MPa and 4.09 MPa, respectively, which is mainly due to the increase in axial yarn content and the decrease in the proportion of braiding yarns. At the same time, the gap between the performance of composites of Type B and C gradually increases, which may be mainly because the axial yarn reduces the bending degree of braiding yarns in composites of Type B, making the bearing capacity improved, while the bending of braiding yarns in composites of Type C almost does not change with axial yarn variation. No matter the axial yarn condition, the modulus of composites of Type A is greater than that of the other two topologies, which may be due to the smaller bending degree and more consistent morphology leading to more uniform deformation under compression. When the braiding angle is 30°, the compressive strength and modulus of the composites with these three topologies are close, regardless of the axial yarn condition, which is mainly due to the weak bearing capacity of the braiding yarns at large braiding angles. At this time, the change in the braiding yarn morphology has little effect on the mechanical properties. In general, when the braiding angle is small and there is no axial yarn, the fabric topology has a significant impact on the longitudinal compressive properties, and the differences in compressive strength and modulus decrease with the increase in braiding angle. The difference in the compressive properties of composites of Type A and B, which are sensitive to axial yarn, decreases with the addition of axial yarns and the increase in size.

## 5. Conclusions

In this paper, the longitudinal and transverse compressive properties of the 3D stepwise rotary braided composites are studied by mechanical tests. Several valuable conclusions can be drawn as follows:

(1) The fabric topology and axial yarn condition affect the yarn morphology in the composites, and the effect of these on the compressive properties depends on the braiding angle.
(2) The fabric topology does affect the compressive property when the braiding angle is small, which makes it a non-negligible factor to be considered.
(3) For composites with large braiding angles, the addition of axial yarn significantly improves the compressive strength, which more than doubles. However, the compressive modulus has an extremum at certain braiding angles, which indicates that the size of axial yarns should be properly selected according to specific demands.
(4) The axial yarn condition has little effect on the transverse compressive property for composites of Type A with a large braiding angle, and it is interesting that the composites with a small braiding angle and no axial yarns perform like linear strengthening materials.

This study provides data and rules for the design of 3D braided composites manufactured from the stepwise rotary braiding process through experimental analyses. Future studies will focus on analyzing the failure mechanism and constructing a predictive model.

## Figures and Tables

**Table 1:** Parameters of the carbon fiber.

Type	Tensile Strength	Tensile Modulus	Density	Elongation	Filament Diameter
TZ700S	4900 MPa	230 GPa	1.8 g/cm^3^	2.1%	7 μm

**Table 2:** Properties of the matrix.

Type	Density	Compressive Strength	Compressive Modulus
JC-02 (100:85)	1.19 g/cm^3^	113.24 MPa	2.54 GPa

**Table 3:** Geometric parameters of test samples.

Fabric Structure	Pitch Length (mm)	Width (mm)	Thickness (mm)	Fiber Volume Fraction (%)	Density (g/cm^3^)	Transverse Compression
A-0-20°	3.1	8.51	8.70	43.66	1.44	Yes
A-0-30°	2.0	8.89	9.03	44.51	1.44	No
A-0-40°	1.6	8.97	9.04	47.96	1.47	Yes
A-12K-20°	3.4	8.81	8.70	56.39	1.51	Yes
A-12K-30°	2.1	9.01	9.15	57.03	1.52	No
A-12K-40°	1.7	9.42	9.44	58.99	1.52	Yes
A-24K-20°	3.7	9.54	9.51	60.56	1.55	Yes
A-24K-30°	2.4	9.90	9.88	59.89	1.54	No
A-24K-40°	1.8	10.24	10.21	60.97	1.54	Yes
A-36K-20°	4.1	10.47	10.58	59.55	1.54	Yes
A-36K-30°	2.6	10.84	10.85	59.76	1.53	No
A-36K-40°	2.0	11.15	11.15	60.75	1.53	Yes
B-0-20°	3.1	8.63	8.54	43.70	1.44	No
B-0-30°	2.0	8.98	9.03	44.78	1.44	No
B-12K-20°	3.4	8.75	8.78	55.60	1.50	No
B-12K-30°	2.1	9.02	9.06	58.28	1.52	No
B-24K-20°	3.7	9.54	9.50	59.53	1.54	No
B-24K-30°	2.4	9.81	9.92	60.75	1.55	No
C-0-20°	3.1	8.59	8.51	44.14	1.43	No
C-0-30°	2.0	8.97	9.05	46.10	1.45	No
C-12K-20°	3.4	8.72	8.77	56.71	1.49	No
C-12K-30°	2.1	9.02	9.06	58.24	1.53	No
C-24K-20°	3.7	9.55	9.52	59.31	1.54	No
C-24K-30°	2.4	9.81	9.91	59.63	1.54	No

**Table 4:** Longitudinal compressive properties of samples.

Fabric Structure	Compressive Strength (MPa)	Standard Deviation (MPa)	Compressive Modulus (GPa)	Standard Deviation (GPa)
A-0-20°	223.05	18.24	19.36	0.12
A-0-30°	109.31	2.01	12.35	0.64
A-0-40°	100.17	1.15	9.78	0.59
A-12K-20°	333.06	13.85	23.99	0.73
A-12K-30°	251.69	10.52	19.91	0.12
A-12K-40°	235.06	3.79	17.02	0.16
A-24K-20°	428.95	10.72	25.27	0.32
A-24K-30°	342.24	3.05	21.71	0.49
A-24K-40°	319.77	13.79	18.16	0.08
A-36K-20°	447.55	14.54	22.50	0.11
A-36K-30°	369.03	5.87	19.76	0.44
A-36K-40°	363.70	11.27	18.42	0.07
B-0-20°	164.96	3.69	16.14	0.45
B-12K-20°	297.16	11.36	20.88	0.85
B-24K-20°	433.04	10.63	24.20	0.31
B-0-30°	104.56	1.09	12.59	0.58
B-12K-30°	252.84	4.56	19.3	0.35
B-24K-30°	361.34	5.50	21.47	0.38
C-0-20°	158.51	10.87	17.68	0.96
C-12K-20°	288.00	7.28	21.49	0.6
C-24K-20°	398.78	3.54	21.47	0.29
C-0-30°	102.80	2.39	12.19	0.53
C-12K-30°	251.07	5.03	19.38	0.35
C-24K-30°	332.93	8.07	21.62	0.35

**Table 5:** Transverse compressive properties of samples.

Fabric Structure	Compressive Strength (MPa)	Standard Deviation (MPa)	Compressive Modulus (GPa)	Standard Deviation (GPa)
A-0-20°	120.67	1.47	3.76	0.02
A-12K-20°	156.60	3.80	4.19	0.03
A-24K-20°	167.53	0.45	4.35	0.02
A-36K-20°	163.53	1.59	4.42	0.01
A-0-40°	105.33	0.97	3.63	0.08
A-12K-40°	120.50	4.21	4.21	0.11
A-24K-40°	118.67	0.37	4.40	0.05
A-36K-40°	114.07	3.57	4.25	0

## Data Availability

The original contributions presented in this study are included in the article. Further inquiries can be directed to the corresponding authors.
